# Real world data on symptomology and diagnostic approaches of 27,840 women living with endometriosis

**DOI:** 10.1038/s41598-021-99681-3

**Published:** 2021-10-14

**Authors:** Kerstin Becker, Klaas Heinemann, Bruno Imthurn, Lena Marions, Sabine Moehner, Christoph Gerlinger, Marco Serrani, Thomas Faustmann

**Affiliations:** 1 ZEG Berlin, 10115 Berlin, Germany; 2 Department of Reproductive Endocrinology, University of Zurich, 8001 Zurich, Switzerland; 3 Department of Clinical Science and Education, Karolinska Institutet, Södersjukhuset, 118 83 Stockholm, Sweden; 4 Statistics and Data Insights, Bayer AG, 13553 Berlin, Germany; 5 Gynecology, Obstetrics and Reproductive Medicine, University Medical School of Saarland, 66421 Homburg, Saar Germany; 6 Bayer AG, 13353 Berlin, Germany

**Keywords:** Signs and symptoms, Reproductive disorders

## Abstract

Endometriosis is a chronic disease that requires a suitable, lifelong treatment. To our knowledge, the **Vi**sanne **P**ost-approval **O**bservational **S**tudy (VIPOS) is to date the largest real-world, non-interventional study investigating hormonal management of endometriosis. We describe women’s experiences of endometriosis in the real world by considering their symptoms and the diagnostic process in their healthcare setting. Overall, 27,840 women were enrolled from six European countries via networks of gynecologists or specialized centers. Of these, 87.8% of women were diagnosed based on clinical symptoms; the greatest and lowest proportions of women were in Russia (94.1%) and Germany (61.9%), respectively. Most women (82.8%) experienced at least one of the triad of endometriosis-associated pain symptoms: pelvic pain, pain after/during sexual intercourse, and painful menstrual periods. The most frequently reported endometriosis-associated symptoms were painful periods (61.8%), heavy/irregular bleeding (50.8%), and pelvic pain (37.2%). Women reported that endometriosis impacted their mood; 55.6% reported feeling “down”, depressed, or hopeless, and 53.2% reported feeling like a failure or having let down family/friends. VIPOS broadens our understanding of endometriosis based on real-world data by exploring the heterogeneity of symptoms women with endometriosis experience and the differences in diagnostic approaches between European countries.

**Trial registration:** ClinicalTrials.gov, NCT01266421; registered 24 December 2010. Registered in the European Union electronic Register of Post-Authorisation Studies as number 1613.

## Introduction

Endometriosis is an estrogen-dependent, chronic, progressive inflammatory disease, histologically characterized by the growth of endometrium-like tissue located outside the uterine cavity^1–4^. The disease is estimated to affect 10–15% of women of reproductive age in the overall populations, however the precise prevalence is not known due to misdiagnosis and delays in diagnosis^4–6^. Endometriosis has a significant impact on physical, sexual, psychologic, and social health, caused by numerous pain symptoms, infertility, pregnancy complications, and psychological distress^6–8^.

Although surgical options have traditionally been a mainstay of treatment, recommendations, guidelines, and general consensus are increasingly moving toward empirical therapy^4,9–12^. Current first- and second-line medical treatments available for endometriosis-related pain include non-steroidal anti-inflammatory drugs and hormonal therapies, such as oral contraceptives (OCs); however, limited clinical trial evidence supports the effectiveness of OCs in treating endometriosis-associated pain^4,10,12–16^. Approved medications include gonadotrophin-releasing hormone (GnRH) analogs, which have proven effectiveness but are associated with clinically relevant side effects and high cost that limit their long-term use^4,9,11–13,17^.

Real-world evidence from observational studies is increasingly relevant and supplements data from randomized controlled trials to improve patient outcomes. In the real-world setting, the long-term risks or benefits and rare adverse events associated with therapeutic interventions can be assessed, as can patient adherence^18^.

There is a paucity of data on real-world outcomes in the management of women with endometriosis, as most studies were conducted in single centers, enrolled small numbers of women, and had limited follow-up periods^19,20^. Furthermore, there are limited long-term data on the safety and tolerability of progestin use in women with endometriosis under real-world conditions, particularly with regard to depressive symptoms, low mood, and bleeding disturbances^21,22^.

Here, we report the baseline data from women enrolled in the **Vi**sanne **P**ost-approval **O**bservational **S**tudy (VIPOS). Dienogest 2 mg/day (Visanne; Bayer) is an oral progestin approved for the treatment of endometriosis in Europe and several other regions worldwide^23^ and is one of the treatments prescribed by gynecologists in VIPOS. To our knowledge, VIPOS is the largest real-world, non-interventional study to date for hormone treatment of endometriosis. This report aims to broaden the understanding of the disease and its management across Europe by describing real-world data on women’s baseline characteristics at study enrollment.

## Results

In total, 27,840 women were enrolled through 1012 gynecologists or specialized endometriosis centers across six European countries (Germany, Switzerland, Russia, Poland, Ukraine, and Hungary). Women could be receiving one of the approved or non-approved (off-label) treatments for endometriosis.

### Study population

The mean overall age at study enrollment was 32.9 years (standard deviation [SD] 9.0), with the lowest age reported by women in Hungary (27.6 [7.6]) and the highest reported by women in Russia (36.1 [8.1]) and Ukraine (37.0 [7.9]). The mean overall body mass index was 23.5 kg/m^2^ (SD 4.3 kg/m^2^), and individual country-specific values were similar. Baseline characteristics and demographics of the study population are shown in Table 1.

**Table 1:** Baseline patient characteristics and demographics of women enrolled in VIPOS. ^a^Patients from Switzerland are not presented here as a subcategory owing to low patient numbers (*n* = 74).

Characteristic	Germany *n* = 1887	Poland *n* = 1179	Russia *n* = 13,159	Hungary *n* = 8992	Ukraine *n* = 2547	Overall *N* = 27,840^a^
Age, years (SD)	31.5 (9.9)	31.8 (8.3)	36.1 (8.1)	27.6 (7.6)	37.0 (7.9)	32.9 (9.0)
BMI, kg/m^2^ (SD)	24.1 (5.3)	22.7 (3.8)	24.2 (4.0)	22.0 (3.7)	24.8 (5.0)	23.5 (4.3)
**Prior pregnancy, %**						
Yes	49.8	54.1	65.6	32.1	84.1	54.8
No	50.2	45.6	34.4	67.9	15.9	45.2
Missing	–	0.3	–	< 0.1	–	< 0.1
**Time from onset to diagnosis, %**						
< 1 year	24.2	48.9	78.7	75.4	47.4	69.6
≥ 1 year	20.8	22.7	9.9	13.8	15.0	13.0
Missing	55.0	28.4	11.4	10.8	37.6	17.4
**Surgical diagnosis, %**						
Yes	38.1	34.5	5.9	12.0	15.0	12.2

*BMI* body mass index, *SD* standard deviation.

### Diagnosis of endometriosis

**Figure 1:**
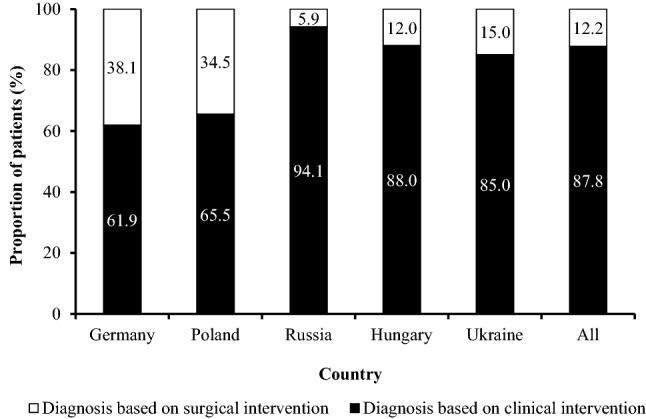
Diagnosis based on clinical symptoms and surgical intervention by country. Patients from Switzerland are not presented here as a subcategory owing to low patient numbers (*n* = 74).

Overall, endometriosis was diagnosed in 87.8% of women based on clinical symptoms (Fig. 1). Diagnosis based on clinical symptoms was most frequently reported in Russia (94.1%), followed by Hungary (88.0%). In contrast, surgical-based diagnosis was most common in Germany (38.1%) and Poland (34.5%). Overall, 69.6% of women were reported to have received diagnosis of endometriosis within the first year after the occurrence of their first endometriosis symptoms.

### Medical treatments

Of the approved treatments prescribed, 11.7% of women received dienogest, 9.3% of women received GnRH agonists, and 3.2% of women received danazol. Non-approved medications for the treatment of endometriosis were prescribed to 62.9% of women receiving combined hormonal contraceptives, 12.4% of women receiving other progestins, and 0.5% of women receiving other non-approved medications for the treatment of endometriosis.

### Disease symptoms and impact on mood before treatment

**Figure 2:**
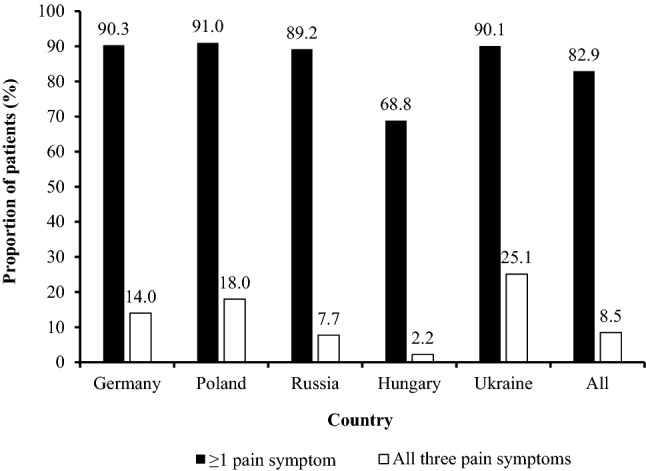
Patients reporting at least one of three selected pain symptoms (pelvic pain, pain during/after intercourse, and painful periods) and all three symptoms, by country. Patients from Switzerland are not presented here as a subcategory owing to low patient numbers (*n* = 74).

Overall, 82.9% of women reported experiencing at least one of the triad of pain symptoms typical of endometriosis (pelvic pain, pain after/during sexual intercourse, and painful menstrual period) via questionnaire at baseline. This was reported by more than 68% of women in each country investigated; the highest percentage was reported by women in Poland (91.0%) and the lowest by women in Hungary (68.8%) (Fig. 2). Only a small proportion of women (8.5%) reported experiencing all three pain symptoms typical of endometriosis; the highest percentage was reported by women in Ukraine (25.1%) and the lowest by women in Hungary (2.2%).

**Figure 3:**
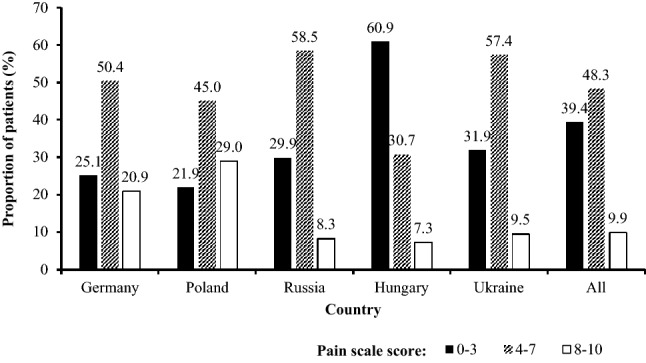
Patient self-reported pain as assessed by numeric scale where 0‒3 indicates mild pain, 4‒7 indicates moderate pain, and 8‒10 indicates severe pain. Patients from Switzerland are not presented here as a subcategory owing to low patient numbers (*n* = 74).

Before treatment, 39.4%, 48.3%, and 9.9% of women reported their endometriosis-related pain as mild (0‒3), moderate (4‒7), and severe (8‒10) over the preceding 4 weeks, respectively, using a numeric pain scale of 0 (no pain) to 10 (unbearable pain) (Fig. 3). Pain rated as mild was most commonly reported by women in Hungary (60.9%), as moderate in Russia (58.5%), and as severe in Poland (29.0%).

The most frequently reported endometriosis-associated symptoms were painful periods (61.9%), heavy/irregular bleeding (50.8%), and pelvic pain (37.2%) (Table 2). The highest proportions of women reporting pelvic pain (60.2%) and heavy/irregular bleeding (62.5%) were in Ukraine, whereas the highest proportion of women reporting painful periods was in Germany (73.7%).

**Table 2:** Proportion of patients who experienced endometriosis-associated symptoms at study enrollment. ^a^Patients from Switzerland are not presented here as a subcategory owing to low patient numbers (*n* = 74).

Symptom, % of patients	Germany *n* = 1887	Poland *n* = 1179	Russia *n* = 13,159	Hungary *n* = 8992	Ukraine *n* = 2547	Overall patients^a^ *N* = 27,840
Painful periods	73.7	68.3	63.9	52.5	72.0	61.9
Heavy/irregular bleeding	42.7	54.1	49.1	51.5	62.5	50.8
Pelvic pain	45.5	55.5	40.9	21.0	60.2	37.2
Tiredness/weakness	30.0	39.5	25.0	24.4	39.9	27.2
Pain during/after sexual intercourse	27.9	35.6	29.9	14.7	40.8	26.1
Difficulty conceiving/infertility	12.5	15.9	21.8	4.4	19.2	15.1
Constipation or diarrhea	15.8	23.5	11.7	10.1	18.8	12.7
Pain when passing urine	8.6	10.5	15.0	4.7	6.8	10.3
Pain during bowel movement	12.5	13.5	13.9	2.4	8.4	9.6

^a^Data obtained from patients receiving dienogest 2 mg/day, or other approved or non-approved medications for the treatment of endometriosis.

Women reported on their mood at baseline via questionnaire, and how they felt endometriosis (and treatment, if received before study enrollment) impacted them by using a scale to indicate how often they experienced a given mood or feeling. Ratings were: 0 (never), 1 (rarely), 2 (sometimes), 3 (often), and 4 (always) (Table 3). Overall, 55.6% of women reported feeling down, depressed, or hopeless (scale values ≥ 1), with the highest proportion in Poland (69.9%) and the lowest in Hungary (49.9%). In total, 53.2% reported feeling like a failure and having let down family (scale values ≥ 1), with the highest proportion in Russia (59.3%) and the lowest in Germany (38.1%).

**Table 3:** Proportion of women reporting impact of endometriosis (and endometriosis treatment, if received) on mood. ^a^Patients from Switzerland are not presented here as a subcategory owing to low patient numbers (n = 74).

Mood, % of patients	Germany *n* = 1887	Poland *n* = 1179	Russia *n* = 13,159	Hungary *n* = 8992	Ukraine *n* = 2547	Overall patients *N* = 27,840^a^
Feeling down, depressed, hopeless at all	63.3	69.9	55.3	49.9	64.0	55.6
Feeling like a failure and having let down friends/family at all	38.1	41.9	59.1	50.5	48.4	53.1
Feeling happy or optimistic about the future at all	82.8	91.0	89.8	94.5	93.6	91.2

^a^Included women reporting scale values of ≥ 1 to indicate how often they experienced a given mood or feeling, with 0 (never), 1 (rarely), 2 (sometimes), 3 (often), and 4 (always).

A large proportion of women (91.4%) reported feeling happy or optimistic about the future (scale values ≥ 1) via questionnaire at baseline, with the highest proportion from Hungary (94.6%) and lowest from Germany (83.0%).

The given percentages represent all women who reported an impact on mood (scale values > 0).

### Study end/outcome

By the end of follow-up, 798 women received long-term treatment (≥ 15 months) with dienogest 2 mg/day. Overall, 309 women received treatment with dienogest for 15–23 months, 222 women received treatment for 24–35 months, 100 women received treatment for 36–47 months, and 167 women received treatment for ≥ 48 months.

## Discussion

Endometriosis is a recognized chronic condition for which an approved effective, well-tolerated, long-term treatment is still an outstanding need. Real-world studies have investigated key areas in the field of endometriosis, including trends in incidence of endometriosis over time, patient and disease characteristics in clinical practice, clinical management trends, and associated diseases^24–26^. Additionally, real-world studies have explored clinical and patient-reported outcomes in routine clinical practice^18,27^.

VIPOS was a non-interventional, observational study conducted between 2010 and 2018 that aimed to broaden the understanding of endometriosis and its management across Europe by describing the real-world user populations of hormonal endometriosis treatments. Of note, women self-reported medical and gynecologic information, thereby providing valuable country-specific and long-term patient-reported data. Here, we explored the range of symptoms that women with endometriosis experience and the diagnostic process they undergo in their local healthcare setting.

Diagnostic delays of 6‒10 years are commonly reported for endometriosis^28–30^. Delayed referral by healthcare practitioners is a key cause, possibly resulting from confounding symptoms, misdiagnosis, trivialization of women’s experiences, and normalization of symptoms^28,30–32^. Real-world studies have also explored the roles of specialist access, cultural influences, socioeconomic status, misdiagnosis, and inappropriate screening as contributory factors in this delay^6,25,31,33^, whereas a lack of disease awareness is also considered a barrier to prioritizing timely diagnosis, especially in young women^32^. In contrast to the published literature, most women enrolled in VIPOS received their diagnosis of endometriosis within 1 year of the appearance of first symptoms. Although the reasons for this remain unclear, most women included in VIPOS were already receiving specialty care, representing a selection bias introduced by the study design. Diagnostic delays caused by lapses of time between the occurrence of first symptoms and referral to specialist care may not have been captured here, which could have contributed partly to the earlier diagnoses observed. Given that our findings are in contrast to those of previous reports on the typical length of diagnostic delays in endometriosis, additional confirmatory investigations are necessary to revise this widely acknowledged phenomenon.

The aim within the healthcare community should certainly be to raise awareness and knowledge of endometriosis among general medical practitioners (including general practitioners, GPs), facilitate early recognition of endometriosis, and address barriers leading to diagnostic delay. For example, in countries where GPs provide basic gynecologic care and are responsible for referral to specialist medical care, GPs may benefit from targeted education. In a publication^32^ involving semistructured focus groups of 43 GPs in the Netherlands, many GPs reported limitations in their knowledge about endometriosis and the endometriosis training they received. Furthermore, almost all GPs considered endometriosis to be a rare disease, and a few expressed uncertainty as to where to find appropriate literature^32^. Greater collaborations between gynecologists and other healthcare professionals is therefore important and should include development of joint guidelines on indications, empirical treatment, referrals, and secondary diagnosis of endometriosis^32^.

In VIPOS, most diagnoses of endometriosis were based on the presentation of clinical symptoms than by surgical means. This indicates appropriate uptake of national guidelines in countries that recommend the use of medical diagnosis, and a shift toward clinical diagnosis in countries such as Germany and Poland, where national guidelines recommend a surgical approach^4,34,35^. International guidelines outlining empirical or initial treatment of symptoms before surgical approaches have also contributed to the observed trend toward medical diagnosis^3,4^, alongside the increased availability of imaging modalities (ultrasound and magnetic resonance imaging). Despite this, other guidelines and literature suggest that clinical diagnosis is still inconsistently applied in medical practice^36^. Practitioners should feel empowered to make clinical diagnoses of endometriosis early and without an invasive procedure, as there is potential for early diagnosis and treatment to relieve women’s pain, avoid central sensitization and pain persistence, prevent infertility, and change the trajectory of women’s lives^36^. Indeed, endometriosis can be suspected without surgical exploration, and medical treatment can be safely prescribed without histologic validation of the disease^37^, and can confirm diagnosis when effective. Therefore, a combination of patient interviews and clinical examinations should be sufficient to enable practitioners to identify women suspected of having endometriosis and who may require imaging assessment^37^, with the effect of appropriate medical therapy also assisting in this identification.

Changes in country-specific behaviors, culture, and reimbursement challenges may also influence this shift in diagnostic approaches. For example, it has been suggested that cultural influences likely affect the clinical presentation of endometriosis^38^ and can lead to differences in conceptualization and reporting of pain, and health-seeking behavior. Furthermore, differences in healthcare experiences, expectations, and efficiencies can impact women’s reporting of symptoms^39^. Race and ethnic background can also affect the provision of healthcare at all levels, which can thereby influence access to care, specialist referral, diagnosis, and treatment^32,38^. Future investigations into the disparity in country-specific rates of clinical diagnosis of endometriosis observed in VIPOS would be interesting, particularly focusing on whether potential barriers and facilitators to the timely diagnosis of endometriosis, such as patient and physician behaviors, can be identified.

Women in VIPOS most commonly reported heavy/irregular bleeding, painful periods, and pelvic pain, followed by tiredness/weakness. In addition, most women experienced at least one of three pain symptoms typically associated with endometriosis (pelvic pain, pain after/during sexual intercourse, and painful menstrual bleeding), and more than 50% of women overall reported an impact on mood due to endometriosis. These observations add to the existing literature on the diverse symptomology of endometriosis and the impact of endometriosis on quality of life^40–42^.

The endometriosis-associated pain symptoms reported in VIPOS are likely already well established in routine clinical questioning of women suspected with endometriosis. Unsurprisingly, the most commonly reported symptoms leading to a diagnosis of endometriosis may be dysmenorrhea and pelvic pain^42^, which form part of the classical triad of endometriosis-associated symptoms on which clinical questioning is traditionally based. Additionally, 50.8% of women experienced heavy/irregular bleeding and 27.2% experienced tiredness/weakness, which has recently been identified as an underestimated symptom of endometriosis^43^. Surprisingly, only 8.5% of women in VIPOS experienced all symptoms of this triad, which is lower than reported elsewhere^42^. These striking reports of symptoms challenge the importance of the symptom triad in the diagnostic questioning of endometriosis. We propose the need for greater awareness of the various and less “traditional” symptoms of endometriosis to ensure a more comprehensive patient-doctor dialogue that may encourage women to seek healthcare and empower them to vocalize their experiences. Albeit inconsistent, there is a correlation between pain severity and endometriosis disease severity^44–46^; however, the severity of endometriosis in women enrolled in VIPOS could not be investigated, as disease staging data was not collected.

VIPOS provides insights into the current treatment choices for endometriosis made by physicians in the real world. Women were most commonly prescribed off-label use of combined hormonal contraceptives (62.9%), such as OCs; although clinical evidence supporting the efficacy of OCs is limited, they are frequently used to treat endometriosis due to their general safety, affordability, and the comfort and familiarity associated with both their use and prescription^15^. Dienogest was prescribed to 11.7% of the women; it is one of few approved medical therapies indicated for endometriosis, and has been studied extensively, demonstrating favorable safety and efficacy in women with endometriosis by improving dysmenorrhea, premenstrual pain, dyspareunia, and pelvic pain, and decreasing the duration of menstrual bleeding and size of endometriomas^47–52^. Other prescribed treatments included GnRH agonists and danazol, although associated side effects restrict their use^4,51^.

From our understanding, VIPOS is the largest real-world non-interventional study of the medical treatment of women with endometriosis, reporting data from 27,840 women with endometriosis across European countries. Detailed questionnaires provided comprehensive assessment of each woman’s gynecologic, medical, and endometriosis history, in addition to their mood, lifestyle, and education. VIPOS raise awareness of how frequently women experience the different symptoms of endometriosis, thereby alluding to the need for more informed clinical questioning considering “non-traditional” symptoms that women may experience. VIPOS also highlights overall adherence to diagnosis by clinical means rather than by surgical interventions. Limitations of VIPOS include the country-specific variability in the number of women and treatment centers enrolled. Given the observational study design of VIPOS, results must be carefully interpreted and consideration given to the fact that residual confounding or bias cannot be entirely eliminated, which may lead to limitations in inferring causation^18,53–55^.

## Conclusions

VIPOS represents a real-world account of the management of endometriosis in specific European healthcare settings through its reporting on the wide heterogeneity of symptoms and the differences in diagnostic approaches that women with endometriosis experience. In particular, the data presented from 27,840 women in this study suggest that clinical diagnosis forms a key element of the medical management of endometriosis in most healthcare settings studied. This supports the need for an open dialogue between physicians and their patients, which should include comprehensive patient interviews designed to capture the spectrum of diverse symptoms that women with endometriosis may be afflicted with. While further study is required to strengthen these findings, the data from VIPOS are supportive of the recent paradigm shift toward clinical diagnosis in the field of endometriosis.

## Methods

The methodology of VIPOS has previously been reported^54,56–59^. Briefly, VIPOS was a prospective, observational, long-term cohort study conducted between 2010 and 2018, enrolling women using various forms of hormonal treatment for endometriosis. Women were enrolled in Germany, Switzerland, Russia, Poland, Ukraine, and Hungary. The number of women enrolled in Switzerland (*n* = 74) was insufficient for separate country analysis, and data obtained from these women have been included only as part of the overall data presented here. In addition, a small proportion of women (*n* = 22) were prescribed a treatment for endometriosis that consisted of more than one hormonal medication concomitantly; they also were included in the overall data set for the description of baseline characteristics but are not described in detail.

The primary objectives of VIPOS were to evaluate the safety of dienogest 2 mg/day compared with other hormonal treatments in the routine clinical practice setting, with a focus on the occurrence of anemia induced by cyclical bleeding disturbances and requiring medical treatment, clinically relevant depression (new or worsening), and drug discontinuation due to treatment failure, as defined by loss/lack of efficacy or an adverse drug reaction. The primary results will be published elsewhere.

Women initiating a new hormonal therapy regimen for endometriosis and willing to participate were eligible for inclusion, while those not willing to participate in a long-term follow-up or with language barriers were not eligible. Hormonal treatments could be approved or not approved (off-label) for the treatment of endometriosis; dienogest 2 mg/day, GnRH analogs, danazol, combined hormonal contraceptives (most commonly combined OCs), other progestins (e.g., dydrogesterone, progestin-only pills, levonorgestrel-containing intrauterine system), and other treatments (hormonal replacement therapy, unknown allocation of concomitant treatments).

To ensure that study participation was not considered necessary for treatment, physicians discussed the study only after the women received their treatment prescription. Enrolled women received a questionnaire at baseline relating to their personal health status and potential risk factors. They self-reported data on their medical and gynecologic history, endometriosis-related symptoms, diagnosis timelines, disease impact on mood, treatment, and lifestyle and educational status. Each woman’s physician completed a subsection of the baseline questionnaire, which included details on the type of endometriosis diagnosis (i.e., based on clinical symptoms or surgical confirmation [direct visualization at laparoscopy and/or histologic assessment of excised endometriosis], with any additional tests, including ultrasound and magnetic resonance imaging) and their recommended prescription and surgical history for disease management. Women were followed up at pre-specified time points, which were 6, 12, 24, and 36 months after baseline and, depending on the date of enrollment, 48, 60, 72, and up to 84 months after baseline. In order to minimize the loss-to-follow-up rate, a multi-faceted, four-level process was utilized: this process has been described and implemented in other studies^54,56–59^. Level one activities included mailing follow-up questionnaires and sending reminders (letters or phone calls), when necessary. If no response was received, level two activities were implemented, which involved multiple attempts to phone women and/or named contacts provided during the study. In parallel, searches in national and international directories and social networks were carried out (level three activities). Finally, formal address inquiries were conducted, where possible (level four activities), to enable interviewers to visit women’s homes or send registered letters with confirmation of receipt.

The ethics information for the VIPOS study has been published previously^55^. The study was approved by one independent ethics committee/institutional review board at each country, where required. In Germany, the Ethics Committee of the Berlin Medical Association approved the study, and in Hungary this was done by the Scientific and Research Ethics Committee of the Medical Research Council. In Switzerland, only one large endometriosis center (Inselspital Bern) took part in the study, and a positive vote from Swissmedic was obtained for this hospital. No ethical approval for non-interventional, observational studies was required by law in Poland, Russia, and Ukraine. Each woman provided written informed consent before participating in the study. The study was conducted in accordance with the Guidelines for Good Pharmacoepidemiology Practices issued by the International Society for Pharmacoepidemiology (2008), the Good Epidemiological Practice-Proper Conduct in Epidemiologic Research statement issued by the International Epidemiological Association European Federation (2007), the European Network of Centres for Pharmacoepidemiology and Pharmacovigilance (ENCePP) Code of Conduct for Scientific Independence and Transparency (2010), and the ethical principles based on the Declaration of Helsinki. The study was prospectively registered in the EU PAS as number 1613 on October 21, 2010, and received an ENCePP seal. In addition, this study was registered on CinicalTrials.gov (NCT01266421) on December 24, 2010.

### Research involving human participants and/or animals

*Statement of human rights* All procedures performed in studies involving human participants were in accordance with the ethical standards of the institutional and/or national research committee and with the 1964 Declaration of Helsinki and its later amendments or comparable ethical standards.

### Statement on the welfare of animals

This article does not contain any studies with animals performed by any of the authors.

### Informed consent

Informed consent was obtained from all participants in the study.

## Data Availability

The datasets generated during and/or analyzed during the current study are available from the corresponding author on reasonable request.
